# Genomic screening for monogenic forms of diabetes

**DOI:** 10.1186/s12916-018-1012-z

**Published:** 2018-02-20

**Authors:** Leslie G. Biesecker

**Affiliations:** Medical Genomics and Metabolic Genetics Branch, National Human Genome Research Institute, National Institutes of Health, Bethesda, MD USA

**Keywords:** Type 2 diabetes mellitus, Maturity-onset diabetes of the young, Gene panel testing, Genome sequencing

## Abstract

Adult-onset, or type II diabetes mellitus (T2DM) has a complex genetic architecture, from hundreds of genes with low penetrance, common susceptibility variants (e.g., *TCF7L2*), to a set of more than ten genes that, when mutated, can cause a single-gene or Mendelian form of T2DM (e.g., *GCK*). It is a clinical challenge to identify patients with the uncommon (2–3%) form of T2DM, typically classified as maturity-onset diabetes of the young (MODY). Bansal et al. (*BMC Med* 15:213, 2017) used a gene panel test approach to test patients with diabetes for single-gene causes of MODY. They found that nearly 2% of younger patients had pathogenic variants in one of seven genes. These data confirm prior studies showing that Mendelian or single-gene MODY can masquerade as garden variety T2DM. The implications of these results for wider general medicine and the future implementation of clinical genome sequencing are discussed.

Please see related article: https://bmcmedicine.biomedcentral.com/articles/10.1186/s12916-017-0977-3

## Background

Most patients with type II diabetes mellitus (T2DM) have the common form of the disease, which genome-wide association studies (GWAS) have associated with numerous common variants [1]. The trait has high heritability [2], and each of these variants, which are common in the population, has additive or other interactive effects. Furthermore, they can be inherited in an individual in combinations that push them above a threshold and lead to disease: so-called polygenic susceptibility. This genetic variation can be assessed using chip-based genotyping [3]. Although the GWAS to find these variants show statistically powerful results, it has been challenging to generate clinically useful polygenic common variant risk scores [4] and thus the clinical utility of many of these variants is modest. In contrast, a subset of patients who appear to have T2DM instead have an uncommon single-gene or Mendelian form of the disease that is caused by one or two rare variants in a single gene. This form of T2DM, which overlaps with the typical T2DM phenotype, is most commonly maturity-onset diabetes of the young (MODY). From a practical, medical management perspective, it is important to distinguish patients with polygenic forms of T2DM from those with single-gene MODY because the management can be different [5, 6]. Therefore, practical and effective ways to distinguish between these two classes of patients are needed.

## The utility of panel testing for MODY genes

In an article published in *BMC Medicine*, Bansal et al. [7] used a candidate gene panel next generation sequencing (NGS) assay to screen patients with diabetes for single-gene causes of MODY. They demonstrated that among patients with early onset (< 40 years) disease, nearly 2% had pathogenic variants in one of seven genes. While it has previously been established that cohorts of T2DM include an admixture of patients with a monogenic form of the disorder [8], this study is notable for several reasons. Firstly, this paper represents something closer to a survey of real-world patients with diabetes, rather than a deeply phenotyped research cohort. Several studies have identified MODY genes in cohorts that were carefully phenotyped to enrich for MODY (see [6] for a review). Such studies are useful for identifying the mutated genes, but not for estimating the wider prevalence. By using relatively relaxed eligibility criteria, Bansal et al. give us a good picture of the admixture of MODY in the larger pool of adult diabetics. Yet, if the yield is 2% among patients in a research study, one can only assume that it is higher in community-based clinical practices. It is important, even if not surprising, that almost 1 in 50 patients in clinical settings with good medical care were undiagnosed. Distinguishing patients with single-gene causes of MODY from the many more patients with garden variety T2DM is difficult, even for expert clinicians [5]. While the textbooks often describe the attributes of ‘classic’ cases, real-world patients have more subtle presentations, making clinical diagnosis difficult. Because they are individually rare, many clinicians are not sufficiently familiar with them to distinguish them from the more common forms of T2DM.

One take-home lesson from this study is that even specialty disease clinics can benefit from a genomic approach to diagnosis. It is unacceptable that 1 in 50 patients with a Mendelian form of MODY are undiagnosed and, again, this fraction is likely higher in the primary care, general practice setting. However, we should not point fingers at the clinicians. Often, when presented with such data, experts will criticize the evidently inadequate diagnostic skills. It is important to remember that the average clinician is midway through their career, having graduated in the middle of their class from an average medical school. They do not specialize in T2DM, which is compounded by the fact that they are extremely busy and have disincentives to contemplate questions such as this. It is understandable that such diagnoses will be missed in many settings and, instead of criticizing clinicians, we must provide them with tools to supplement their diagnostic skills to correctly diagnose such patients. Genomics is a way to do this [9].

Genomic testing and underlying technologies are advancing at a furious pace. It is important to recognize that MODY is primarily caused by rare (and in some cases, novel) variants. These variants are not amenable to detection by genotyping chips and must instead be assayed via sequencing – currently NGS. The cost per nucleotide of assaying genomic DNA for all types of variants is falling rapidly, such that research-based NGS testing now costs approximately USD $1000 for genome sequencing [10]. While clinical testing is much more expensive, its cost also continues to fall and within the next decade, we expect such testing to be widely available at a cost comparable to other commonly used tests, such as magnetic resonance imaging. Only a few years after the Human Genome Project [11] was completed, clinical exome and genome sequencing NGS became available [10], which has been used in the diagnosis of hundreds of thousands of patients with rare genetic disorders. While exome and genome testing are currently too expensive to be used routinely for a disease as common as T2DM, we must anticipate that these falling costs and the ever-increasing clinical utility of this type of testing will expand, even for disorders as common as T2DM. Efficient panel testing may be a bridging technology; indeed, models have shown it to be cost effective as a tool for screening patients with T2DM [12].

Bansal et al. [7] did not use exome or genome sequencing. Instead, they used NGS, implemented as a candidate gene panel, and merged that with a sample pooling strategy to minimize costs. While appropriate for their specific study design, it is unlikely that this is how NGS will be implemented in the future. It will more likely be used for individual genome sequencing. Even if NGS data generation costs continue to decline, dividing the cost of genome sequencing by a diagnostic yield of 2% in early-onset T2DM magnifies the cost of identifying each case 50-fold. Viewed that way, it is hard to imagine how genome sequencing could be cost effective for this single use.

However, this approach to costing sequencing belies its general utility. Indeed, as we move forward, costs of single-gene tests and panels will approach genome sequencing. At this point, the cost-effective strategy will be to perform genome sequencing once, early in the course of many diseases, and to use the data across the genome in numerous ways for many purposes, including T2DM diagnosis, cancer predisposition screening, carrier screening, and pharmacogenetics. In this way, the cost of sequencing for any one of these uses plummets further, again because of the broad potential utility for such sequencing. The data generated by Bansal et al. [7] are one tile of the mosaic of clinical utility and, when combined with many other tiles, will generate a picture of the long-term clinical utility of sequencing.

## Conclusions

Bansal et al. demonstrated the use of an NGS panel test to distinguish the uncommon disease MODY from garden variety T2DM. Such testing, implemented on a wide scale, is likely to be inexpensive, and its application would identify patients with MODY who should be managed distinctly from those with typical T2DM. In this way, a genomic tool can be used to identify patients who otherwise go undiagnosed, and who have high risks of having affected family members, who could also benefit from diagnosis and treatment. It has long been said that genomics is coming to general medicine, in what has generally been assumed to be some far-off, distant future. The data on the genetics of MODY and T2DM make a compelling case that genomics should be used today. It is not some far-off future – it is here.
